# Death as attraction: the role of travel medicine and psychological travel health care in ‘dark tourism’

**DOI:** 10.1186/s40794-021-00149-z

**Published:** 2021-08-11

**Authors:** Irmgard L. Bauer

**Affiliations:** College of Healthcare Sciences, Division of Tropical Health and Medicine, James Cook University, Townsville, QLD 4811 Australia

**Keywords:** Thanatourism, Travel health advice, Travel psychiatry, Travel motivations, Tourist behaviour, Psychological health, Mental health, Travel psychosis, Voyeurism

## Abstract

Still an evolving field in travel medicine, psychological travel health has not yet been linked to tourist products that may affect travellers’ mental wellbeing. Dark tourism, the travel to sites linked to death, atrocities and suffering, is a product that, on the one hand, attracts people with a keen interest in death-related attractions and, on the other hand, may inflict psychological scars. Of particular concern are travellers with undiagnosed or diagnosed mental illness.

This is the first article bringing travel medicine and dark tourism together. Understanding dark tourism is crucial to appreciate the wide variety of potential stimuli leading to anything from amusement to travel-related psychoses. Travellers’ motivations for and emotional responses to visits of ‘dark’ sites provide an important input into individually tailored psychological pre and post-travel health care. Relevant recommendations include suggestions for education, clinical practice and much needed further multidisciplinary research.

## Introduction

‘Holidays in Hell’, in a 2019 Australian weekend magazine [1], reported highly inappropriate visitor behaviour at so-called ‘dark tourism’ sites, a relatively recently studied, fast developing form of tourism whose health impact on visitors and local residents is still awaiting discussion in travel medicine. Death as an outcome of travel concerns this specialty, death as the travel attraction, so far, does not.

The term ‘dark tourism’ was coined in 1996 [2] to represent visitations of sites of death or inhumane acts. It is ‘concerned with tourist encounters with places of death or calamity that have perturbed the public conscience, whereby actual or recreated places of the deceased, horror, atrocity or depravity, are consumed through visitor experiences’ ([3], p307). What is the fascination of death, and what makes people interested in travelling to objects and sites of human tragedy, cruelty and gore? Subsequently, how does the emotional drive to see, and the impact of the experience, affect such travellers, especially those with diagnosed or undiagnosed mental illness? The literature on dark tourism over the last two decades is vast, consisting of complex and comprehensive in-depth discussion and exploration of a wide range of areas within the spectrum of social sciences, psychology and tourism, supported by different philosophical underpinnings. A full discussion is beyond the scope of this paper.

The purpose of this paper is to summarise key points across the breadth of dark tourism providing a baseline for travel medicine to develop pre and post-travel care strategies, as psychological aspects of travel are still underappreciated. This breadth is necessary because individual travellers’ responses to stimuli may present in ways that do not reflect the type and intensity anticipated based on the ‘gore-value’ of a location. After placing the topic in a death-tourism nexus and discussing the changing meaning of death throughout the times, the evolving concept of dark tourism is presented to highlight the supply of destinations. On the demand side, travellers’ motivations and emotional involvement, including selected tentative potential theoretical explanations, allow some insight into the possible mindset of travellers to dark tourism sites.

## Method

Extensively discussed in tourism and social sciences for the last 20 years, the umbrella term ‘dark tourism’ was used in database searches using PubMed, Scopus, Web of Science, ScienceDirect, ProQuest, Google Scholar, as well as grey literature and relevant websites, as any more recent re-classifications still include this phrase. Reference lists of obtained papers yielded further sources. The health literature does not seem to concern itself with the topic: only one very loosely relevant source was obtained through PubMed. Only English-language articles, chapters, monographs and limited media reports were used. All sources were printed and read in their entirety with a focus on 1) the concept of dark tourism and 2) any indication of people’s psychological and emotional involvement in seeking out places of death and tragedy. The same procedure applied to all other topics.

## Death and tourism

The link between death and tourism is multifaceted. Travel medicine’s focus is on preparing the traveller for a healthy trip and safe return home. Nevertheless, deaths of travellers happen during travel or at destinations due to illness, accidents or misfortune, or post-travel from a cause acquired during the trip. Such events may be presented as case studies or as statistics, for example, of national tourists dying overseas [4] or visitors dying at a particular location [5]. A death can be the reason for travel, for example, to attend a funeral or to retrieve the body of a loved one. Death can also be the purpose of a trip (‘death tourism’, suicide tourism’, ‘euthanasia tourism’). Travellers may be seeking the services of agencies to arrange voluntary assisted dying not legally available in their home county [6]. Others travel with the plan to commit suicide at a meaningful site (‘the suicide-bridge’) or a romantic or geographical location of significance. Hotels are also places of death. Apart from natural deaths of guests or long-term residents (often celebrities or the wealthy), misadventure, murder and suicides, some people plan to die in a hotel, with or without the knowledge of management, for a number of reasons and subsequent implications for staff and administration [7]. However, ‘travel and death’ represents a much larger concept.

**Fig. 1:**
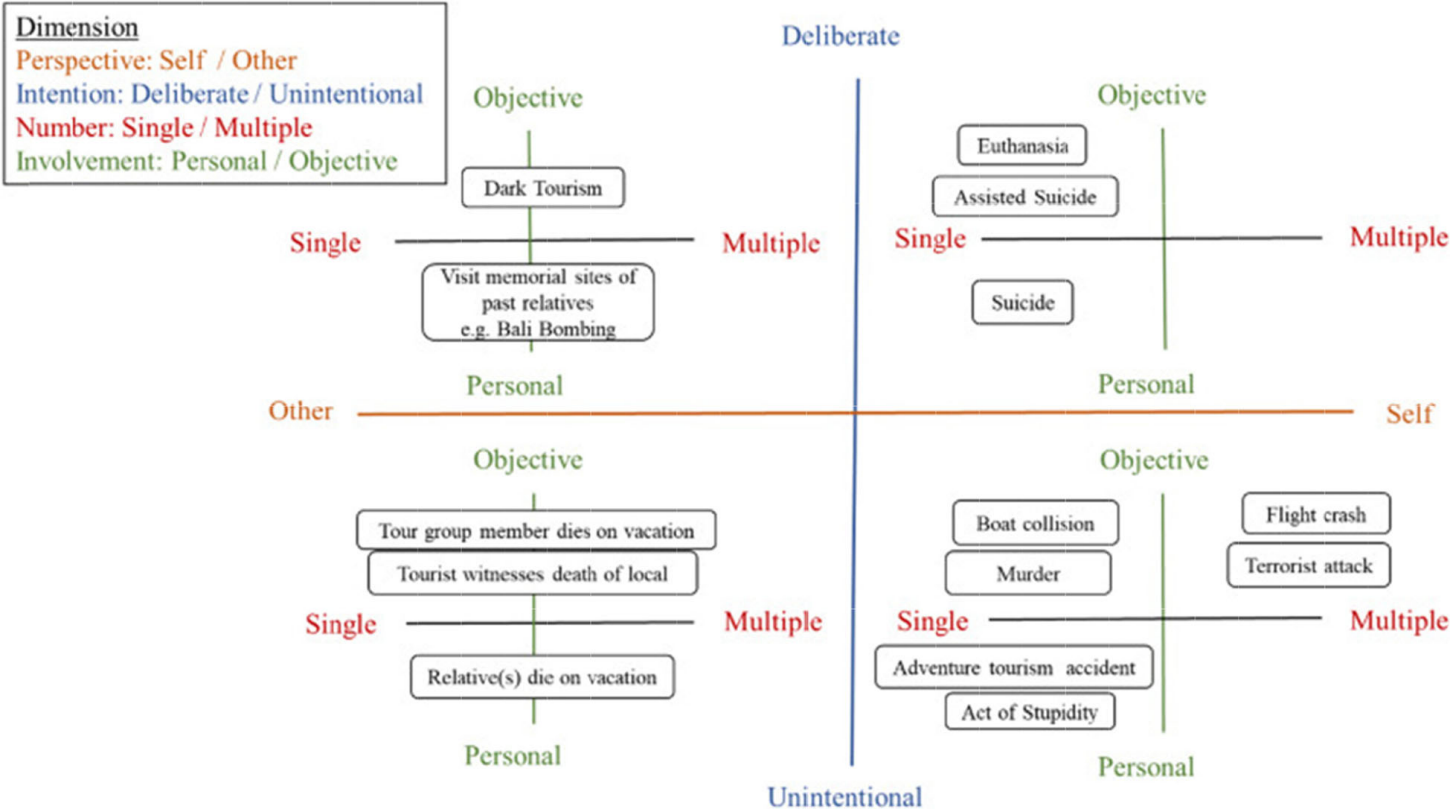
Death-Tourism Nexus [8] (used with permission)

From an anthropological and an existential perspective, Pratt et al. [8] developed a framework representing a death-tourism nexus based on four dimensions (Fig. 1): 1) death perspective dimension (from self to others), 2) death intension dimension (from deliberate to unintentional), 3) death number dimension (from single to multiple) and 4) death involvement dimension (from personal to objective), embedding dark tourism in its appropriate place. This concept and its evolution over time will be presented after, first, exploring the understanding and perception of death throughout history.

## The meaning of death throughout history

Knowledge of the medical aspects of death alone does not provide insight into the spiritual and cultural perceptions of the end of life and, subsequently, death’s potential attraction. Thanatopsis, the contemplation of death of oneself or others in recent or distant times, plays a crucial role in understanding changing philosophical approaches to death. French historian Philippe Ariès’ (1914–1984) categories of historically and culturally evolving ‘death mentalities’ have provided a structure for further development in understanding dark tourism [8, 9] and shall serve here for a summarised overview of the gradually changing meaning of death (in Western society) through the last millennium.

The ‘*tamed death’* of the Middle Ages embodied a strong familiarity with death where dying, death and mourning was not just a private but also a public affair. Predominantly Church-guided through obedience by terror, death – although feared – was part of human existence. Depictions of (religious) death scenes or a set of memento mori, e.g. *danse macabre* (dancing skeletons carrying away the dead), and skulls or hourglasses in people’s homes reminded everybody of the physical and spiritual proximity of death [9].

In the late Middle Ages, shared death slowly shifted to the individual *‘death of self’* with the focus on the individual’s personal responsibility for a life that will ultimately determine the verdict on the final Day of Judgment. Highly visible religious thanatoptic displays reminded of the terror of death [10], its individuality also expressed through personalised tombstones [9]. During the Renaissance, a brief shift to the *‘remote and imminent death’* occurred where death could be brutal, strike at any time and one must fear.

This view was gradually replaced during Romanticism and the Victorian era (late 18th to late nineteenth century) where through the *‘death of the other’*, sadness, loss and separation was expressed in art, poetry and elaborate mourning which, at least for the wealthy, could turn into extravagant public spectacles with processions and ostentatious tombs and mausoleums. Black romanticism had a particular connection to death in its preoccupation with torture, executions, Satan, ghosts, Gothic darkness, ‘where pleasure becomes confused with pain, beauty and horror’ [11]. ‘The Sublime’, the increased pleasure obtained from phenomena that instil terror and awe, is evident in the production of art and literature of the time. This change of attitude to death emerged at a time when religion and superstition made way for science and emerging alternative ideologies [10].

Political and societal changes and medical advances in the twentieth century steadily changed the perception of death (*‘forbidden death’, ‘invisible death’*). This modern death relies on three aspects [9]. First, the medicalisation of death, where religion has been slowly replaced by medical technology, the priest by the doctor, and the home by the hospital where death and dying happens away from public sight, the dying are lied to, and the dead are stored in morgues. Second, the privatisation of mourning ensures that there are no public outpourings of grief; the bereaved are lonely, requiring the service of grief therapists. Third, the deritualisation of death and mourning makes sure that life goes on without major interruption, for example, with timesaving cremations. In short, death has turned from something natural to something pathological. Over the last half century, most Western adults under forty never have stood at a deathbed or been present at the moment of death [12]. Terror Management Theory, based on the view that unconscious fear of death influences everyday life, has been proposed to offer ways of managing attitudes towards death [13]. While the theory has evolved over the decades, it does not yet seem to cater for fear of death manifested in an attraction to horror and death.

Mechanisms of dealing with this absent death through denial or detachment is offered in the consumption of death in popular culture, such as television, movies, music, print, games or jokes [14]. In 1995, the average American 16-year old has seen 18,000 murders on TV [14]. Soaps, reality shows and cartoons contain death as does live news of funerals, wars or atrocities. Death is part of western, war or crime movies; slasher films depict sex, brutalisation and death [15], snuff movies the actual death of an actor on camera. Death themes are in all musical genres from opera to folk, ‘coffin songs’, heavy metal and rap, enhanced by actual early deaths of music icons. Much of the literature contains death as the principal topic while humour and jokes permit the abandonment of good taste by focusing on the body (cannibals, necrophilia) or the person (homicide, last words, executions), even though such humour may be offensive to those grieving [14]. Interestingly, the *Journal of Vampire Studies* enjoyed a re-launch in 2020. The treatment of death as entertainment may symbolise death denial but may also be an expression for a hunger for more insight into death.

These developments led to an extension of Ariès’ framework with the *‘spectacular death’* of today where death is taking centre stage in five different forms [9]. First, the new mediated visibility of death: daily news bring visions of bodies to one’s living room, though, increasingly, some outlets have started to blur ‘offensive’ sights. Wars, terrorist attacks and dying refugees provide a constant supply of death. The exhibition *Body Worlds* by Gunther von Hagens displays plastinated actual human bodies and body parts [16]; televised celebrity funerals allow public participation. Second, the commercialisation of death, as seen in the controversial Benetton advertisement of a dying AIDS patient [17], the withdrawn Hyundai commercial focusing on an attempted suicide [18], or crass souvenirs such as Auschwitz fridge magnets [19]. Third, the deritualisation of death, with the emergence of delightful marketing of funeral parlours [20], colourful party-themed burials with personalised coffins and pop music, and where the celebration of life takes away the mourning for the deceased. Fourth, the palliative care revolution, where power is transferred to patients and relatives to make decisions, to care for the dying rather than to keeping them alive at all cost, where self-determination mirrors a change in worldviews and created a medical speciality. Fifth, death as the topic of academic attention and specialisation. From the 1960s, interest in death and grieving started to evolve [21]; Elisabeth Kübler-Ross’ (1924–2004) ground-breaking book ‘On Death and Dying’ appeared in 1969. Only from the 1990s did death become a major focus of interest in the social sciences [9].

“*Spectacular death”* inaugurated a period in which death is gradually returning from forced exile during “*forbidden death”* and is now something discussed and exposed in public through the media although “*spectacular death”* simultaneously commodifies death and makes it a bizarre object of shallow consumption and entertainment’[9,p.17]. Dark tourism is one avenue to do just that.

## The concept of dark tourism

Dark tourism developed relatively unnoticed by the public until bad behaviour brought it to light in the media. The coining of the term in 1996 [2] started a more systematic inquiry into tourism to death-related locations (= supply of a tourist product) with the first categorisations, such as distinct travel actions [10] or ‘Divisions of the Dark ‘[22] (Table 1).

**Table 1:** Divisions of the Dark [22]

**1**	**Perilous Places**
	a) Towns of Terror - Ghost towns b) Dangerous Destinations - Current war zones, slams (favela tourism), recent tourist massacres (Luxor, Tunisia)
**2**	**Houses of Horror**
	Homes of murderers (OJ Simson), of murders (Gianni Versace) a) Dungeons of Death - Alcatraz, Chateau d’If, Devil’s Island, Changi, Lubyanka HQ, Robben Island, Melbourne City Gaol b) Heinous Hotels (turning jails into hotels) - Obersalzberg
**3**	**Fields of Fatality**
	a) Bloody Battlegrounds; Waterloo, Crimea - La Higuera/Bolivia (Che Guevara) b) Hell of Holocaust - ‘Schindler-Tourism’; remnants of hair, teeth, shoes, spectacles; Danish concentration camp turned into hotel c) Cemeteries for Celebrities - Père Lachaise/Paris - Cappuchin Crypt/Palermo - Killing Fields in Cambodia
**4**	**Tours of Torment**
	a) Mayhem and Murder - Hollywood’s grave line tours (in a hearse) b) The Now Notorious - E.g. Ronnie Biggs
**5**	**Themed Thanatos**
	a) Morbid Museums - Lima: Palace of the Inquisition - Pathology Museums - Graceland, Althorp b) Monuments to Morality - Gruesome outcomes for social deviants, e.g., Ten Courts of Hell/Singapore; Hell House in Denver

‘Thanatourism’ [10] is often used interchangeably, the difference seen by some as thanatourism referring to ancient events visited out of meditative interest and dark tourism driven by commodification of recent events [23], even though the former may be equally commercialised and for a long time. ‘Thanatos’ may have been chosen as more detached and so more acceptable than ‘death’.

The history of travel to gore and death is long. Ancient Rome treated visitors to gladiators ripped apart in the Coliseum. Eleventh century pilgrims to Jerusalem encountered a busy trade in relics and holy water. The Grand Tour to Europe in the 17th to mid-nineteenth century included a large number of attractions based on the Christian death cult, such as relics, bones, part of cadavers, morgues, as well as places of cruelty and death; the educational meditation of one’s own mortality may often have given way to light entertainment. Mark Twain’s trip to Europe and the Holy Land in 1867 offered plenty of ‘must-sees’ [24], though he noted his own reaction as being fascinated by some, ‘disinterested, amused, frustrated and even deeply uncomfortable’ by others [25]. Victorians visited Bedlam (St Mary of Bethlehem Hospital) to watch the cruel ‘care’ of the feeble-minded [26]; the first guided tour in England was allegedly a train ride to a public hanging; others visited the then famous Paris morgue [27]. In retrospect, much of historic travel would be classed today as some form of dark tourism.

However, the original concept proved too general with multiple variables, such as authenticity (authentic vs created [28], passage of time [26, 29, 30] purpose (education, entertainment, remembrance, propaganda), site association (site *of* death vs *about* death [29]), or if a visit is just part of a recreational itinerary instead of a trip’s sole purpose. Another attempt to manage a concept that seemed expanding with every new publication was to use four shades of darkness (or ‘macabre-ness’) as a possible start for a useful framework [31]). Philip Stone’s Dark Tourism Spectrum [32] provides seven categories of dark supplies from the lightest to the darkest dark on a dynamic continuum, a spectrum that has been refined over time and shall be used here with a range of examples to summarise what is an ever-growing, yet still ill-defined, topic.

1. Dark Fun Factories (the lightest shade)
  These commercial entertainments with a high degree of tourism structure provide a sanitised product, allowing the light-hearted viewing of, or ‘morbid gaze’ [33] upon suffering and death in a socially acceptable environment. Examples are the many dungeons visited for a thrill not education, though, perplexingly, a visitor thought it was ‘a great way to get children involved in history’ [[34],p.364].
2. Dark Exhibitions
  These are commercial sites with a tourism structure built on a reflective, educational and commemorative message and often away from the actual site of the event to which they refer. Such exhibitions are museums (often sanitising the content of the event), Gunther von Hagens’ *Body Worlds* [16], the *Catacombe dei Cappucini* in Palermo where since 1599 hundreds of mummified bodies are on display, or the Sedlec Ossuary in the Czech Republic.
3. Dark Dungeons
  These focus on former prisons or houses of justice, authentic or not, with an edutainment component, where tourists favour especially the size of cells, or places of execution. Historic prisons, such as in Melbourne, penal colonies, such as Norfolk Island off Australia, or sites of the recent past, such as Robben Island or Alcatraz [35] may resemble (open-air) museums or theme parks.
4. Dark Resting Places
  Romanticised or macabre, cemeteries and gravesites enjoy great popularity [36], often with guided tours focusing on the celebrated dead, the architecture of mausoleums, or the aesthetic value of sculptures. Famous cemeteries, such as Père Lachaise in Paris (Oscar Wilde, Frédéric Chopin, Jim Morrison), Highgate in London (Karl Marx, George Michael), La Recoleta in Buenos Aires (Eva Perón), or innumerable celebrities in Hollywood’s cemeteries, are becoming highly commercial enterprises (‘fun-tours’) while others, such as Robert Louis Stevenson (Samoa) or Paul Gauguin and Jacques Brel (Marquesas Islands) attract the more intrepid traveller. Others are visited for the spectacle of being embalmed (Ho Chi Minh, Lenin). Human bones and mummified remnants can still be found in the Peruvian desert around Nazca. Bali funerals and Indian ghat cremations attract large numbers of tourists. A visit to displays of self-mummified Buddhist monks (*Sokushinbutsu*) in Japan is recommended for ‘like-generating’ Instagram posts [37].
5. Dark Shrines
  Close to the event, and evident through floral and personal tributes, shrines are usually temporary with little to no tourism infrastructure; ‘grieving’ visitors have no personal relationship with the dead. Over time, such shrines can turn into a commercial spectacle with emerging necessary tourism structures as at Ground Zero [38].
6. Dark Conflict Sites
  These locations, linked to warfare, battlefields and war cemeteries around the world, relate especially to WWI and WWII, such as Verdun [30], Gallipoli [39] or Guadalcanal in the Solomon Islands. While time has passed, visitors will still remember parents or grandparents who had been at war, and there is still enough connection to serve as a place of commemoration. Today, such sites are often commercialised and relay political ideologies and propaganda [40]. Guernica or the ruins of Belchite remind of the atrocities of the Spanish Civil War. Memorials in Hiroshima and Nagasaki [41], Jeju Peace Park in Korea [42] or the Comfort Women Museum [43] both in South Korea remind of atrocities in the Asian region. Regardless of the danger, current conflict zones, e.g. the Middle East, draw interested visitors [44]. Conflicts further back in time (real or in memory), such as the US civil war or the battle of Waterloo, are often ‘commemorated’ in battle re-enactments with a distinct fun component.
7. Dark Camps of Genocide (the darkest shade)
  Perhaps the ‘original’ dark tourism, these sites commemorate places of barbarism, atrocities, catastrophe and suffering throughout the times, guided by a high degree of political ideology or religious connotation, e.g. sites of the Inquisition. Much literature covers the darkest type of attraction, starting with the Holocaust sites of concentration and extermination camps of Auschwitz, Treblinka, Buchenwald and many more [40], and here especially the emotional aspects of such visits [45–50].
  More recently, memorial sites for the 1994 Rwandan genocide attract visitors not only for contemporary atrocities or the displays of bloodstained clothes and actual remains of victims but the opportunity to meet perpetrators in person [51, 52]. Similarly to Auschwitz which is ‘the thing to do’ when in Poland [27], one wonders if the opportunity to view corpses is the main motivation for such visits. Other darkest sites are mass graves around the world, e.g. on the Balkan, or the Memorial of the Nanjing Massacre in China [53]. Sites of barbaric political torture, interrogation and executions are the Villa Grimaldi in Chile, now a *Parque por la Paz* (Peace Park) [54], the Khmer Rouge Tuol Sleng Genocide Museum and the Choeung Ek Killing Fields in Phnom Penh, or Budapest’s House of Terror Museum. The history of slavery is commemorated at not only the destinations, e.g. US plantations, or at the source of finance, management and control, e.g. the Slave Museum in Liverpool [55]. Especially poignant are the sites of incarceration and embarkation of African slaves, such as Ghana’s Slave Castles [56–59] or the idyllic *Ile Gorée* off Dakar/Senegal, where through the ‘Door of No Return’ slaves took their last step on home soil.

The above structure is certainly fluid, and not all current death-related tourist attractions fit neatly. Some relate simply to a site of death, others are abjection-oriented. Sites of death are usually commemorated with some plaque or construction where visitors congregate. There may be a different perception of sites of natural disasters vs man-made atrocities. *Natural disasters* shock due to the large number of casualties, helplessness against forces of nature and where, most of the time, no one can be held accountable. Pompeii/Italy is a classic example; others are the buried village of Te Wairoa/NZ (after the 1889 explosion of Mt Tarawera) [60], or the colliery spoil collapse on a school in Aberfan/Wales 1966. A devastating earthquake in 1970 buried the town of Yungay/Peru, now a vast cemetery, similarly to the site of the 2008 Beichuan earthquake killing 70,000. More recently, the fatal volcanic eruption in December 2019 on Whakaari White Island/NZ created demand to resume ‘fligh-overs’ a mere month after the event [61].

Favourite sites of *man-made events* are murders and assassinations of famous people, such as John F Kennedy in Dallas [2], Gandhi in New Delhi (the final footsteps are marked on the ground), John Lennon in New York, or Che Guevara La Higuera/Bolivia. Unknown victims attract if they were particularly vulnerable, e.g. school-shootings or the PanAm Lockerbie bombing in 1988, or their demise was especially gruesome, such as the Snowtown murders in South Australia, where tortured victims were found in acid barrels in an unused bank [62]. Houses of murders or home of murderers are often destroyed to avoid the thrill-seeking masses. Large-scale accidents attract, such as Pripyat in the Chernobyl exclusion zone [63], the 1987 capsize of *Herald of Free Enterprise* in Zeebrugge, but also locations of celebrity car crashes (James Dean, Marc Bolan, or Princess Diana). Celebrity deaths in hotels allow management to charge premium for such highly sought-after rooms [7], almost as if the surfaces were still contaminated by the air that surrounded the event. Some rooms are attractions, e.g. Oscar Wilde’s room 16 in the Hotel d’Alsace, or Janis Joplin’s Highland Garden Hotel, room 105. Others, such as room 434 at the Beverly Hilton (Whitney Houston), are taken out of rotation to avoid precisely that attraction. No doubt, at some stage, the Wuhan wet markets may become another destination of interest.

Abjection-orientated attractions fall under extreme dark tourism, celebrate dystopia, an imagined post-apocalyptic society of great suffering, and offer tourists the engagement with violence and horror. Charles Manson’s ‘Helter Skelter Tours’ in Los Angeles, or the H.R. Giger Museum in Gruyeres/Switzerland provide visceral closeness to violence [64]. The clearest example are the annual Black Metal festivals in Norway and elsewhere – one remembers the related church burnings and murders in the 1990s – which attract ‘blackpackers’ from around the world. The participation in ritual-like concerts, dousing fermented blood, decaying animals or vomit onto the audience, satanic worship, putrid smells, topics of cutting, self-harm and death, as well as the danger of real violence can arouse an appreciative and expectant audience to some transitory mind zone [65, 66].

The above discussion has demonstrated the enormous breadth of travel destinations linked to death, which will expand with modern media transmitting news of yet more tragedies, hence, potential destinations. Has dark tourism become a superficial label? The Taj Mahal and the Pyramids are tombs and highly commercialised, but are they dark tourism sites? Is travel to one’s mother’s gravesite in another town dark tourism, or the participation in *El Día de los Muertos* in Mexico?

What is dark? It appears that, across cultures and times, there has always been a metaphorical association between light and goodness, and dark and evil [67]. We understand ‘dark’ as disturbing, troubling, weird, morbid or perverse leading to negative emotions and experiences, such as horror, fear, depression or sadness [68], though changes in politics and culture may change the perception of a dark past [32]. Dark tourism contrasts with light holidays (sea, sand, fun), is based on dark deeds (atrocities, murder) and leads to dark outcomes (mood) [69]. However, relating to this topic, darkness as a socially constructed concept is Eurocentric and seems to work only in the English language as translations, e.g. *dunkler Tourismus* or *turismo oscuro,* make little sense [70], and may be incomprehensible, or not applicable at all, to Asian perspectives [41].

When the term was first coined, it was probably not foreseen that, over time, it would become so stretched that it has been criticised as poorly conceptualised [71], loosely defined [68], and theoretically fragile [27], and it ‘oversimplifies a complex multi-faceted and multi-dimensional phenomenon’ ([31], p.220) to the point that it has lost its usefulness [72] and impedes further detailed analysis [69]. There is also the permanent tension with heritage tourism [73] where people may visit for patriotic and personal reasons rather than death and gore [39]. Over time, a number of subcategories have been proposed such as the thanatourism typologies [23] in Table 2.

**Table 2:** Thanatourism Typologies [23]

1	**Horror Tourism**
	Potential to trivialise death through commodification (shocking and humorous) - Chambers of horror - Jack the Ripper
2	**Grief Tourism**
	- Arlington, Graceland, assassination sites
3	**Hardship Tourism**
	- Slavery, slums, prisons, Robben Island
4	**Tragedy Tourism**
	- Accidental or deliberate disasters, - Ground Zero, Pompeii, Hurricane Katrina
5	**Warfare Tourism**
	- Battlefield tours, war museums, battle re-enactments, war memorials
6	**Genocide Tourism**
	- Killing fields in Cambodia, Buchenwald
7	**Extreme Tourism**
	- Public executions, Bali cremations, restaging of Christ’s crucifixion

Further additions are, for example, ‘prison tourism’ [74], ‘phoenix tourism’ visiting sites recovering from disasters, e.g. the 2004 Indian Ocean tsunami or 2005 Hurricane Katrina [75], or ‘drought tourism’ [76]. There may well be the time when the spectrum of darkness may lose its importance and only focus on the darkest end. Of increasing importance, virtual or ‘imaginative’ [26] travel to destinations of death and suffering seems neglected but, with recent travel restrictions, is a worthwhile aspect for investigations. The ‘Philippine Thriller Video’ of 1500 inmates forced to dance for up to four hours to avoid retributions led to the ‘shows’ becoming an actual destination delighting tourists with the spectacle of punishment [77].

Any new field in any discipline evolves over time and re-defines itself. Fun factories may not be part of that future; newly identified facets may become the core issue, the term ‘dark’ may disappear. Such changes should not concern travel medicine. Regardless of which philosophical angle, category, classification or label one uses, in the end, it is the appreciation of the emotional engagement and effect on the traveller that should concern travel health practitioners because the reaction on consumption of horror is subjective, highly individual and unpredictable. Torture instruments in a fun dungeon may shock one traveller whereas another remains detached when visiting a former concentration camp. One may suffer deep sadness reading a commemorative plaque while another finds positive arousal imagining how people died. The next section discusses motivations, emotions as well as some theoretical underpinnings as a starting point for further research into psychological travel health.

## Motivations and emotional engagement – the unsolved puzzle

Dark destinations represent the supply of tourism products for which there is a customer demand. Understanding tourist motivations for, and reaction to, this ‘darkness’ is important when designing appropriate travel health care.

### Motivations

What makes people travel to look at corpses, participate in satanic rituals and criminal actions, take happy snaps at Holocaust sites, or play-act torture and executions? Notoriously difficult to research, evidenced by the gap in neurobiological measures, many reasons prompt ‘dark’ visits, even visiting a cemetery solely for its peaceful atmosphere [78]. On the one hand, personal obligations [45], remembrance, patriotism [39], empathy for victims [73], and education [79] are strong motivators. Most visitors to concentration camps are school classes or young adults [70], although this does not account for individuals’ motives. To learn about violence to prevent future atrocities [80], however, seems unconvincing. Visits also allow personal reflections about one’s own mortality [22, 26, 78]. Others are driven by curiosity [73, 79], a need for novel entertainment [81, 82], attraction to horror and ghoulish titillation [73], celebration of crime, deviance and basic bloodlust [22], indulgence in violence and suffering [68], secret pleasure in terror, violence and the transgressive [11], ‘the morbid thrill of looking at a corpse’ [83], *Schadenfreude*, the pleasure at the misfortune of others [73, 78], or a desire to experience abjection in ritual-like context [66]. Horror, as a source of the sublime, an awe-inspiring aesthetic, can be enjoyed as long as there is a physical or virtual distance to the event. One may find this aesthetic in the act of killing, by a criminal or a butcher [84] as long as one watches from a safe distance, such as tourists aware of the safety of the bus awaiting them at the entrance, or the safety of the travel itinerary. The tourists’ life is not in danger where others have come to a gruesome end. Relatively recent psychoanalytic approaches to tourist experiences may yield more insight in the future [44]. Once at the location, travellers’ emotions are more accessible to investigations.

### Emotional impact

Typically, people go on holidays to have positive experiences. Little is known about the emotional effect on visitors to dark places [47]. Emotional responses are wide-ranging and mirror in part the dark spectrum discussed earlier with more studies conducted at Holocaust sites [46, 49] or when entering conflict-zones [85]. Very little is known about children’s responses [86] travelling with family or in a school class. For many, ‘fun-dungeons’ offer hilarious and amusing entertainment [33, 34, 71]; others experience positive arousal at black metal concerts [66]. However, as experiences are highly individual, disturbing emotions can arise, often unexpectedly, across the entire spectrum. Norm-approved feelings, such as compassion [76], empathy, sorrow, national pride [81] or appreciation of victims [53] are expected of most persons but can be burdensome for the visitor. Negative feelings, such as sadness, horror, grief, fear, disgust, anger, shock, depression, shame and guilt [46, 49, 50, 53, 56, 73, 81] can have a long-lasting effect on visitors that may not be communicated to family, friends or clinicians. Emotional experiences are what is probably most remembered after a trip. From an industry point of view, people may not return if a site was not satisfactorily sad enough; on the other hand, negative feelings can facilitate subsequent positive behaviour [87]. Emotional responses of potential customers are incorporated in product design and marketing by utilising the fact that visual experience of dark and light influences the body-mind interaction. Online presentations of dark tourism products should also include lighter images ‘to reduce tourists’ discomfort and avoid an impact on their physical and mental health’ ([88], p.11). A recent review of psychological responses to horror films [89] offers ideas for further research, some of which lend themselves to investigations in travel medicine. Examples are research into the mechanics of evoking fear and disgust but also coping mechanisms at particular dark tourism sites, or emotional responses to ‘likeable’ victims (vs. villains). Methodological considerations include both qualitative and quantitative methods to elicit feelings, cross-cultural samples to understand responses to death in individualistic vs. collectivistic cultures, and a focus on homogenous dark tourism locations, e.g. exclusively on war cemeteries or slave castles.

### Behaviour at sites

It is still unclear what influences, guides and permits travellers’ subsequent behaviour at dark sites. Numerous examples of insensitive visitor behaviour lead to negative feelings among other visitors and local residents. At the time of writing, visitors to Buchenwald played winter sports on its grounds [90]. Considering the often highly sensitive context in which such flaunts happen, poor comportment causes offense. Much criticism focuses on disrespectful behaviour, laughing, smoking, ‘chilling’ or the all-important selfies at Holocaust sites [45, 48], confirmed by this author when visiting Mauthausen, the site of a relative’s death. Visitors may find cafés and gift shops [59], or public transport close to solemn sites [91] sacrilegious and upsetting. It has been suggested that dark sites should be ‘interpreted as symbolic; that in the face of death, tourism sanctifies life’ ([91], p.50) and so exuberant behaviour is justified, although few visitors or residents may be so forgiving.

Responsible travel, here minimising impact on residents regardless of visitor behaviour, applies not only to developing countries. Old suffering, created by the original atrocity, can be reopened, intensified and prolonged by tourists [80, 92]; cold-case tours violate residents’ sensitivities. The production of crass souvenirs, tasteless photo props and other commercialisation of murders saw one local entrepreneur driven out of town [62]. The psychological scars of ‘atrocity-hosts’ are not well understood. To appreciate dark tourism completely, the community must be involved [62]. Travellers may feel disquiet at their (respectful) presence, others, perhaps *post fact*, at their inappropriate ‘goofing about’ and employ neutralisation techniques, presented later in this section. Solid evidence for the horror-pleasure paradox still lacking, the ‘pornography of death’ and voyeurism are some potential concepts of interest.

### Tentative theoretical rationalisations

As early as 1719, Abbé Jean-Baptise Du Bos wondered why some people find pleasure in representations of horror which would be extremely unpleasant to witness directly, and may relish in *Schadenfreude*, but he was not able to solve this ‘Du Bos Paradox’ [93]. Pornography, traditionally, refers to the private and tabooed enjoyment of sexual representations for one’s own sexual gratification. With a shift in worldviews in the twentieth century, a social acceptance of sexuality and a decline in religious beliefs, the previously mundane death slipped into the realm of titillating subjects. ‘Pornography of death’ [94] in the 1950s referred to the increasing depictions of death in the media and forensic records. Later on, until 2003, photographs of corpses taken in a morgue studio served as gallery art, eliciting in the viewer a ‘value judgement that condemns the invisibility of death’ [12]. This consumption, similar to *Body Worlds*, creates a *frisson*, an aesthetic shudder, ‘equated with the orgasm of the voyeur that marks the crossing of forbidden thresholds – and pleasure in that crossing’ [12].

Voyeurism is the gaining of sexual pleasure from watching others undress/ed. or in a sexual act – considered a deviation from normal behaviour [95] – or of enjoyment from seeing others in pain or distress [96]. While in the first case, the watched is unaware, in the latter this activity is mainly overt, as in dark tourism. The desire to travel to a place that is socially constructed as forbidden, and subsequently dangerous to enter, makes a trip ‘dark’ [97]. Freud’s *scopophilia*, the pleasure of looking, may clash with a feeling of shame about this desire, yet, this drive is neutralised by devising morally acceptable reasons for visiting [92].

### Neutralisation techniques

A number of techniques are employed to justify norm-contradictive behaviour, familiar to travel medicine practitioners as not taking malaria pills or eating unsafe food. Neutralisation (pre-behaviour/pre-travel) and rationalisation (post-behaviour/post-travel) have originally been attributed to delinquency but apply to any real or perceived socially deviant behaviour. The original five techniques [98, 99] easily apply to travellers: 1) denial of responsibility: action is beyond the traveller’s control, e.g. in organised tours, 2) denial of injury: visit/behaviour, even though perhaps not a good thing, did not do great harm, 3) denial of the victim: the object of the visit deserves to be stared at, e.g. public punishment, 4) condemnation for the condemner: travellers focus on those who disapprove of such visits, 5) appeal to higher loyalties: social norms within a group of travellers (e.g. ‘blackpackers’) override norms of society. Over time, further techniques were added [100], such as ‘metaphor of the ledger’: the traveller claims to not normally doing anything like this, ‘defence of necessity’: a visit is justified, as there is a real need, e.g. religious, emotional drive, ‘denial of the necessity of the law’: social rules are questioned, ‘everybody does it’: traveller does nothing extraordinary, ‘claim of entitlement’: the traveller has a right to gaze, or ‘postponement’: the traveller regrets ‘I wasn’t thinking’. Different neutralisation techniques were applied by tourists engaging in the controversial climbing of Uluṟu, a site of religious and cultural significance to the Aṉangu people of Central Australia [101]. The climb has been closed permanently in 2019.

## The role of travel medicine and psychological travel health care

Rows upon rows of black and white photos of mutilated bodies can leave long-lasting psychological scars with a traveller; others may suffer from nightmares after viewing torture instruments in a fun factory. Some may be moved to tears in a slavery museum while a visit to a genocide location provides others with a thrilling experience.

Considering the interest in travellers’ mental health in the general medical, health and psychiatric literature, travel medicine’s concerns seem still quite limited with few publications. The aetiology of travel-related psychiatric symptoms is extensive [102]. Incomplete grieving processes may also lead to later, more regressive expressions [21]. However, a link to potential impacts of dark tourism is still missing. Since tourists can travel with (un) diagnosed psychiatric issues, not detected or revealed in a pre-travel consultation, emotional reactions to dark objects or sites may trigger various types and degrees of mental disturbance. One can make interesting connections to a number of syndromes allegedly triggered by visits to high emotional valence sites (religious, cultural, spiritual or aesthetic), where excitement, stimulation, unconcise fantasies and many other possible causes, such as jetlag or alcohol possibly trigger a travel-related psychosis [103]. Three such psychologic conditions, where a return home can be the best treatment, are mentioned here. The *Paris Syndrome* [104] refers to a profound disappointment of Japanese tourists due to an over-romanticised expectation of the city. The *Stendhal Syndrome* describes acute onsets of psychic discomfort, including hospitalisations, due to an overpowering emotion triggered by the beauty of art objects in Florence [105].

There are compelling links between the *Jerusalem Syndrome* where some tourists are hospitalised after being struck by psychotic episodes based on religious delusions [106, 107] and the potential impact of death-related emotions in dark tourism. Clinical experience based on the referral of 1200 tourists and 470 hospitalisations over 13 years with severe location-generated mental problems, led to the proposal of three types of the syndrome [107]. While speculative due to lack of research, there are potential parallels to dark tourism (Table 3).

**Table 3:** The *Jerusalem Syndrome* classification by type and subtype [107] (used with permission), applied to dark tourism

Type	Reason for coming to destination	Travel Mode	Pre-existing psychiatric illness	Subtypes
Type I	Psychiatric ideation, need to accomplish mission	Usually alone	Documented psychiatric history: schizophrenia or bipolar illness	I(i) identification with character **(murderer, torturer, victim)** I (ii) identification with idea **(satanic rituals, righting wrongs of killings)** I (iii) magical ideals concerning health/sickness/healing possibilities **(relics, holy water)** I (iv) problems with family **(unresolved grieving, family disapproval of interest in horror)**
Type II	Curiosity plus strange (non-psychotic) thoughts or mission	Usually in groups, sometimes alone	Non-psychotic mental disorders: personality disorders; fixed ideas	II(i) appears in groups **(aims at changing society; Gothic appearance and unusual behaviour)** II (ii) individual **(aims at correcting displays, sites, type of veneration, behaviour)**
Type III	(Jerusalem syndrome discrete type) Regular tourists	With friends or family; often as part of organised tour	No previous psychiatric history or psychopathology	no subtypes: all cases characterised by highly predictable well described clinical stages **(e.g. anxiety, obsessive behaviour, need to display particular behaviour at site, vocalised display of views on horror or death, no visual or auditory hallucinations)**

Suggested examples of dark tourism applications appear in bold font

Consequently, Types I and II of the syndrome would be seen in travellers with underlying psychiatric disorders including diagnosed psychotic conditions (Type I) or non-psychotic mental disorders (Type II). Type III, the ‘pure’ or ‘unconfounded’ form of the syndrome because not linked to psychopathology [107], may occur unpredictably and resolves usually within a week with full recovery. Without further research, individuals resembling Types I and II, and travellers who display mental distress in anticipation of a trip or on conclusion, are potentially the first to focus on in travel health consultations. Considering the high religious significance of the hajj, it is perhaps surprising that similarly strong religious feelings did not enter the discussion of psychiatric illnesses among pilgrims [108], many of whom will have waited years in high anticipation to reach Mecca.

## Limitations and recommendations

The section on dark tourism in this review focuses on English-language literature in tourism and social science as none could be located in any health field, and on Western perspectives as this is where dark tourism originated. It also summarises greatly the often highly abstract concepts; more detail can be found in dedicated volumes [109, 110]. Local language literature from destinations related to death and suffering would yield additional supporting or rejecting views, especially when related to local residents’ perceptions of thrill-seeking tourists. The available travel medicine literature on psychological travel health care highlights its scarceness and the increasing call for inclusion into traditional travel medicine. The large body of general medical and psychiatric literature referring to travel-related mental problems still needs to be reviewed, ideally co-authored with a psychologist or psychiatrist, to develop this section in travel medicine. Regardless of those limitations, recommendations can be made for education, clinical practice and further research.

### Education and clinical practice

A general lack of basic psychiatric preparation in travel medicine [102, 111] makes its inclusion in courses, examinations and conferences necessary [112]. A coughing traveller is not referred to a pulmonologist, nor one with diarrhoea to a gastroenterologist. Similarly, travel clinicians should be able to conduct pre and post-travel psychological assessments or ask the right questions if the chosen destination may have detrimental psychological effects on a vulnerable traveller.

In the same way as travellers raise red flags when advising their participation in a ‘drug retreat’ [113], at least extreme dark tourism destinations should trigger further questions. For travellers with a diagnosed mental illness, cooperation with a mental health team is essential; cooperation with psychologists and psychiatrists will also improve practitioners’ skills in dealing with travel psychology/psychiatry. Educational information for travellers with mental illness and their caregivers is available online (e.g. [114]) and the International Association for Medical Assistance to Travellers (IAMAT) provides relevant factsheets [115–119] which serve well as a basis for conversations during consultations. The International Society of Travel Medicine’s (ISTM) interest group ‘Psychological Health of Travellers’ is in an excellent position to provide education not only to health professionals but to transport staff on planes, cruise ships, and trains, and to police and others who may respond to affected travellers on location or in transit. The ISTM ‘Responsible Travel Group’ guidelines need expansion to include appropriate behaviour at sites to avoid giving offense to other visitors and local residents. Regional travel medicine societies around the globe should provide similar assistance to clinicians and residents at dark tourism destinations.

### Further research

The inter-disciplinary domain of tourism research usually excludes health because little effort has been made in the past by health professionals to collaborate when topics clearly suggest meaningful collaboration [120, 121]. Studies into visitor motivation and emotional responses have served interpretation and site management purposes, not the tourists’ mental health. The field of possible topics is wide open. While social science researchers are better equipped to study emotions in detail, travel health practitioners can build on this body of knowledge and explore further the link between travellers’ emotions and appropriate care if needed, starting with case studies and qualitative investigation. More multidisciplinary research is needed on tourism motivation [11, 73], an ideal topics for cooperation when recruiting travellers consulting a clinic. Obsession with death in psychology [122, 123] serves as a starting point applying tools such as the Death Obsession Scale [124, 125]. Galvanic skin responses measure psychological arousal. Multimodal Discourse Analysis [126], based on a multimodal framework for analysing websites [127], not only allows the assessment of dark tourism websites regarding information, creation of motivations and expectations, and expected behaviour, which may help diagnosis relating to a particular visited site, it is generally useful in travel medicine for assessing web based health information.

Ethical issues from the travellers’ perspective and dilemmas during a visit are under-researched as are lasting experiences, especially of children and young people [73]. We need to know more about particular visitor groups, such as war veterans returning to sites of combat, descendants of African slaves returning to West Africa, or family members to a site of mass destruction to address their needs appropriately. Much more research is needed to understand the experiences of local residents whose lives were impacted directly by atrocities the tourist come to see, or who are inconvenienced by tourist hordes looking for a particular ghoulish location [8, 73].

## Conclusion

This paper is the first to bring dark tourism and travel medicine together to explore potential pathways for developing high-quality psychological travel health care. Dark tourism, a relatively recently described concept, is still evolving and expanding. Regardless of its fluid status, the impact of travel to death-related locations, be they fun parks or places of pure evil, on individual travellers’ mental health has not yet made an appearance in travel medicine. Travellers may come home with a fever, which will be attended to; their potential psychological scars remain invisible. The effect of confronting death and violence during travel, incidental or sought, on travellers with diagnosed or undiagnosed mental illness requires particular attention. Urgently needed education and training, clinical guidelines and a plethora of research are required now to prepare travel medicine practitioners to detect, assess, treat or refer travellers, and cooperate with psychologists or psychiatrists as required pre, during and post travel. Travel medicine has expanded continually over the last 25 years. Its inclusion of psychological travel health is long overdue, and the voices calling for remedy are getting louder.

## Data Availability

Not applicable.

## Abbreviations

IAMAT: International Association for Medical Assistance to Travellers
ISTM: The International Society of Travel Medicine
